# A316 ACUTE NECROTIZING PANCREATITIS COMPLICATED BY PULMONARY EMBOLISM: A CASE REPORT AND REVIEW OF LITERATURE

**DOI:** 10.1093/jcag/gwad061.316

**Published:** 2024-02-14

**Authors:** C Shamatutu, R Barclay

**Affiliations:** Medicine, The University of British Columbia, Vancouver, BC, Canada; Medicine, The University of British Columbia, Vancouver, BC, Canada

## Abstract

**Background:**

Acute pancreatitis (AP) is an inflammatory condition associated with local and systemic complications. Severe pancreatitis can include the development of peripancreatic fluid collections and necrotizing pancreatitis (NP). Vascular complications are one of the causes of morbidity and mortality in patients with AP. Splanchnic thrombosis has been well described in AP and can been seen in up to 24% of patients. Extra splanchnic vessel thrombosis, including pulmonary embolism (PE), is rare.

**Aims:**

Describe a case of acute NP with peripancreatic fluid collection complicated with bilateral PE and review the relevant literature.

**Methods:**

We performed a literature review for AP complicated by PE.

**Results:**

Case: A 60-year-old man presented to the hospital with one day of abdominal pain, nausea and vomiting. His medical history was significant for obesity with a BMI of 40. He was not on any medications. He did not drink alcohol. On exam his heart rate was 58, blood pressure 149/94 mmHG, oxygen saturation was 95% on room air, he was afebrile. His abdomen was tender in the epigastric region. His labs revealed a white blood cell count of 14 x 10^9^/L, lipase ampersand:003E 3,000 U/L, Bilirubin 40 umol/ L, AST 613 U/L, ALT 527 U/L, GGT 394 U/L, ALP 94 U/L, Creatinine 159 mmol/L. CT abdomen/pelvis revealed cholelithiasis without choledocholithiasis or biliary dilation. The pancreas was inflammed with necrosis throughout. There were two peripancreatic fluid collections (60mm x 41mm, and 148mm x 89mm). He was diagnosed with acute necrotizing pancreatitis thought secondary to gallstones. He was fluid resuscitated, started on antibiotics and unfractionated heparin (UFH) for deep vein thrombosis (DVT) prophylaxis. His oral intake remained low. Imaging on day 30 showed a large walled off necrotic collection (25cm x15cm). Vascular strucutres were patent. On day 36 he underwent endoscopic ultrasound guided drainage via lumen-apposing metal stent (LAMS). On day 43 he developed new tachycardia. CT chest revealed bilateral pulmonary emboli (Image 1). Doppler ultrasound revealed bilateral occlusive thrombus in the calf veins. He was treated with therapeutic UFH.

Literature review: We identified 10 case reports describing AP complicated by PE. The age amonst reports was variable (21 to 68). 4 cases reported additional thrombosis outside of the pulmonary vasculature. No cases which described splanchnic venous thrombosis prior. 3 cases reported pancreatic necrosis. The use of DVT prophylaxis was not reported. There is one case of PE 2 days after LAMS insertion in a patient with known thromboembolic disease.

**Conclusions:**

PE is a rare complication of AP. Novel to this case was the recent deployment of a LAMS, the significance of which is unknown to date. Improved documentation of variables in future reports is needed to further characterize this event.

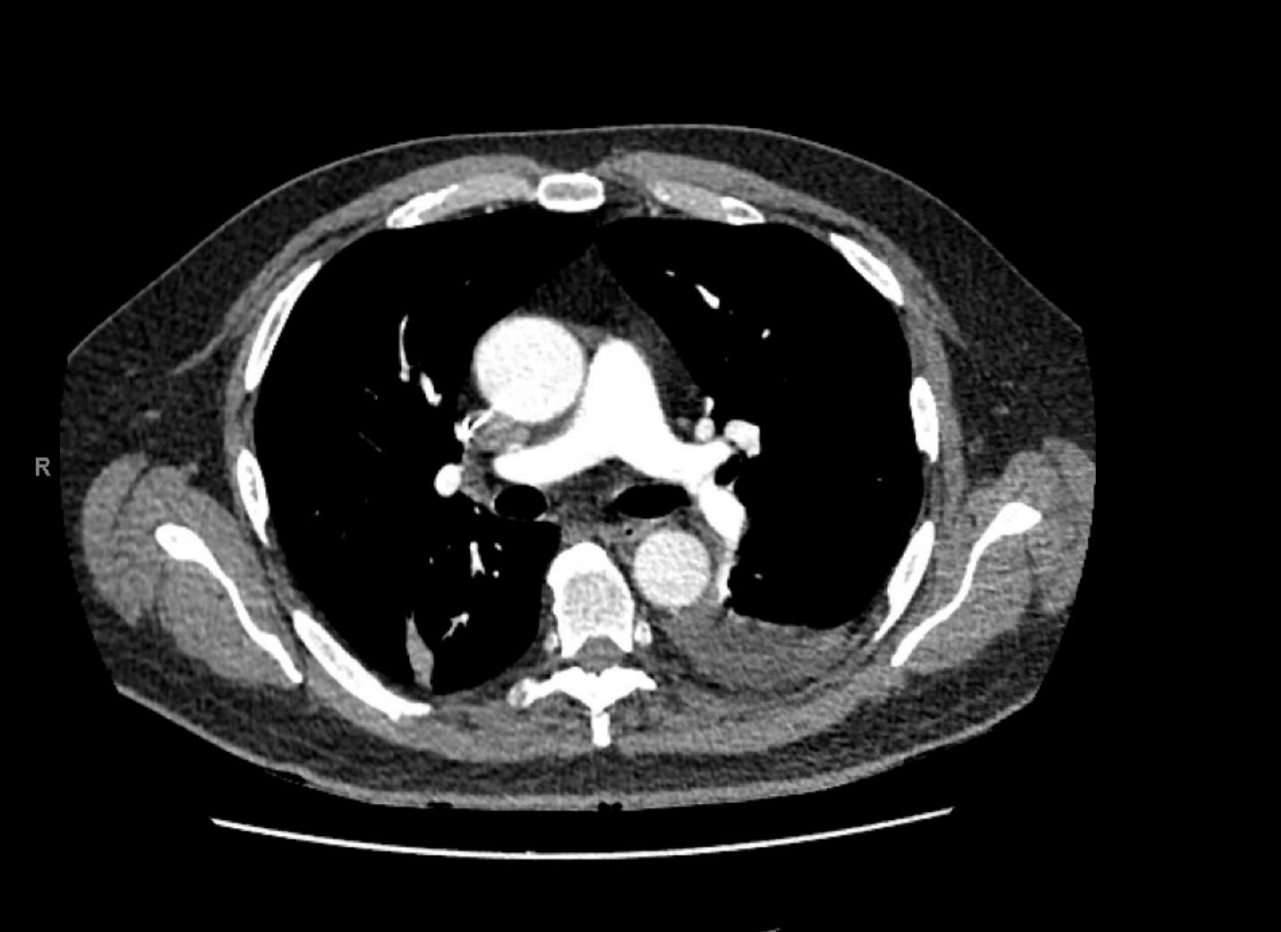

Image 1: Acute Pulmonary Embolism.

**Funding Agencies:**

None

